# Secrecy Analysis and Error Probability of LIS-Aided Communication Systems under Nakagami-*m* Fading

**DOI:** 10.3390/e23101284

**Published:** 2021-09-30

**Authors:** Ricardo Coelho Ferreira, Michelle S. P. Facina, Felipe A. P. de Figueiredo, Gustavo Fraidenraich, Eduardo Rodrigues de Lima

**Affiliations:** 1 Department of Communications, Faculty of Electrical and Computer Engineering, State University of Campinas, Av. Albert Einstein 400, Campinas 13083-970, SP, Brazil; r228003@g.unicamp.br (R.C.F.); mfacina@decom.fee.unicamp.br (M.S.P.F.); 2 Instituto Nacional de Telecomunicações, Santa Rita do Sapucaí 37540-000, MG, Brazil; felipe.figueiredo@inatel.br; 3 Department of Hardware Design, Instituto de Pesquisas Eldorado, Campinas 3083-898, SP, Brazil; eduardo.lima@eldorado.org.br

**Keywords:** large intelligent surfaces, 6G, bit error and secrecy outage probability, Nakagami fading, Von Mises distribution

## Abstract

Large intelligent surfaces (LIS) are a new trend to achieve higher spectral efficiency and signal-to-noise ratio in mobile communications. For this reason, this paper proposes metrics to analyze the performance of systems with multiple antennas aided by LIS and derive the spectral efficiency, secrecy outage probability, and bit error probability in an environment with Nakagami-*m* distributed fading. In addition to an eavesdropper, there is a single-antenna user, an array of antennas at the transmitter side and the possibility of a direct link between transmitter and receiver. This study assumes that the LIS performs non-ideal phase cancellation leading to a residual phase error that follows a Von Mises distribution, and shows that the resulting channel can be accurately approximated by a Gamma distributed SNR whose parameters are analytically derived. From these formulas, it is possible to evaluate the effect of the strength of the line-of-sight link by varying the Nakagami parameter, *m*.

## 1. Introduction

Large Intelligent surfaces (LIS) are a promising technology for beyond fifth-generation (B5G) systems, given the number of papers emphasizing their advantages, whether compared to relays [1] or even when used to enhance the power of millimeter wave technologies [2]. Furthermore, reflecting signals with extreme precision and without power consumption can reduce the interference and improve the signal-to-noise ratio at the receiver, especially when the direct path between transmitter and destination is weak and needs to be strengthened.

In addition, known as large reflecting surfaces, they have recently been studied as a solution for different modulation schemes and communication channels. Their performance metrics show their significant potential for mobile communications. For example, Yang et al. [3] proposed a transmission protocol to reduce the channel estimation overhead when adjacent cells share the same reflection coefficients. In addition, optimization methods are used to allocate the transmit power and maximize the achievable rate in an orthogonal frequency division multiplexing (OFDM) scheme under frequency-selective channels.

In [4], Basar presented a mathematical framework to obtain the signal-to-noise ratio and derive the symbol error probability of an LIS-aided communication system, with or without knowledge of the channel phases. The author also proposed an access point sending signals directly to the users aided by a LIS system. Wymeersch et al. [2] emphasized that, although there are already other techniques for high frequencies (0.1 to 1 THz), these technologies are limited by multipath propagation and obstacles presented in the environment. In this case, LIS can control the physical propagation environment, decrease energy consumption, and simplify location and mapping systems, creating a line-of-sight (LoS) path between transmitter and receiver.

In [5], the authors presented solutions for the adjustment of the LIS elements’ phases, which optimizes the channel capacity and the precoder applied on the transmitter side. Elbir et al. [6] developed a deep learning framework to obtain the channel state information (CSI) in a massive multiuser MIMO system aided by a LIS. The authors estimated each user’s composite channel and the direct path through a convolutional neural network whose inputs are the received pilot signals. Lin et al. [7] performed channel estimation by applying Lagrange multipliers and a dual ascent-based scheme iteratively. They also found a closed-form solution for Cramer–Rao lower bounds and proposed a method that improves the accuracy of the classical least-square method. Taha et al. [8] presented an energy-efficient architecture where all the LIS’s elements are passive except for a few distributed active elements that are arranged in a non-uniform manner. The reflector array applies deep learning models to obtain the optimal matrices of phase shifts.

Although an LIS is usually a panel of reflectors physically organized in planar shapes, Hu et al. [9] proposed alternative structures with a three-dimensional spatial configuration with spherical surfaces. In addition to broader coverage, they have a more straightforward positioning system when compared to the conventional planar arrays.

LIS must be large in far-field communications to compete with classic massive MIMO systems and compensate for multipath propagation and electromagnetic interference. Besides that, optimizing the phase shifts associated with each element of the LIS is a great challenge. Therefore, in [10], Najafi et al. proposed an optimization method based on the physical modeling of the propagation and clusterization of a thousand reflectors into small subsets, also known as tiles. Based on concepts from radar communications, they modeled the impact of each tile on the overall channel, calculated the associated electric and magnetic fields, and showed that it is possible to optimize the operation of the LIS to maximize some quality of service (QoS) criteria.

On the other hand, Garcia et al. [11] focused on near-field environments and established a relation between the array size and the Fresnel zones. The punctual approximation of the scattering characterization presented dependence on the second and third-order moments of the distance. On the contrary, for far-field, the dependence is given for the fourth power. Kishk et al. [12] employed some stochastic geometry tools to analyze the effect of the large-scale deployment of LIS on the performance of cellular networks in the presence of blockages surfaces. They established a relation between the density of LIS panels and blockages.

Mukherjee [13] explores the idea of integrating LIS with mobile edge computing (MEC) technology that intends to leave computing involved in processing the received signal to a cloud server and describes how these technologies can mutually benefit and create a framework competitive for 6G. Finally, Malandrino et al. [14] analyze the possible benefits of using intelligent and reflective surfaces to increase the privacy and security of mobile communications through secrecy rate, considering that passive eavesdroppers are involved in the system, in addition to legitimate users.

In addition to the works related to optimal estimation and power control in transmission systems aided by LIS, it has become a trend to compute the channel’s capacity in the face of eavesdroppers. The question to answer is: “Does such a system offer the physical layer security that prevents an intruder from receiving a signal not intended for him?” The secrecy outage probability metric can answer this question since it means the probability that the instantaneous secrecy capacity is less than or equal to a given capacity threshold. Below are some works that, like ours, are concerned with information security in systems assisted by LIS.

### Related Works

For the case of Gaussian distributed channels and considering parameters such as the distances between devices and the number of LIS elements, Yang et al. [15] derived closed-form expression for the secrecy outage probability (SOP) assuming that the LIS uses CSI to implement the phase shifting perfectly. In its turn, Trigui et al. [16] assumed a more realistic model in which there are errors caused by phase quantization. By leveraging Fox’s H transforms, they obtained exact SOP expression under the assumption that many reconfigurable elements of LIS and channels were distributed according to the Rayleigh distribution.

On the other hand, Ai et al. [17] demonstrated the potential of improving secrecy with LIS aid under different scenarios where a passive eavesdropper is attempting to retrieve the transmitted information: a vehicular-to-vehicular and a vehicular-to-infrastructure. Makarfi et al. [18] showed how the source power, eavesdropper distance, the number of LIS elements, the source-to-relay distance, and the secrecy threshold affect the secrecy capacity and SOP when the vehicular source uses an LIS as an access point.

Following the perspective of the physical layer security, this paper analyzes the secrecy outage probability of a LIS-assisted system in which *K* antennas at BS transmit simultaneous signals to only one user. As shown in [19], the overall fading coefficient is approximately gamma distributed, even for small values of *N* and *K*, but only when the Nakagami-*m* fading channels have m=1 (Rayleigh distribution). The reasoning is extended here to more general scenarios in which *m* assumes arbitrary values. To the best of the authors’ knowledge, this is the first analysis covering both channels with and without a line of sight. The derived closed-expressions for bit error probability (BER) and SOP allow us to conclude that it is possible to evaluate the system performance and design it without performing several Monte Carlo simulations that would be computationally costly in a scenario with multiple antennas and multiple reflectors. The use of the gamma approximation is investigated for a more general scenario, in which it is possible for a line of sight path to exist or not in each one of the intermediate channels (i.e., paths between the transmitter and the LIS, and between the LIS and the user).

In contrary to our previous work [19], this study focuses on secrecy analysis and extends the system model to near-field scenarios. The presence of an unwanted eavesdropper link is a realistic consideration since the information leakage becomes increasingly worrisome, especially for banking, corporate, and government communications in addition to demonstrating the validity of the proposed bit error probability approach when analyzing environments with Nakagami-*m* fading.

The paper is organized as follows: Section 2 presents the system model and the initial equations that based the formulation of the problem, while Section 3 presents the closed-form expressions for spectral efficiency, BER, upper bound, and SOP. Finally, Section 4 demonstrates the validity of the proposed analytical expressions through Monte Carlo simulations and Section 5 presents the final considerations. Demonstrations and mathematical deductions are presented in Appendix A.

## 2. System Model

**Figure 1:**
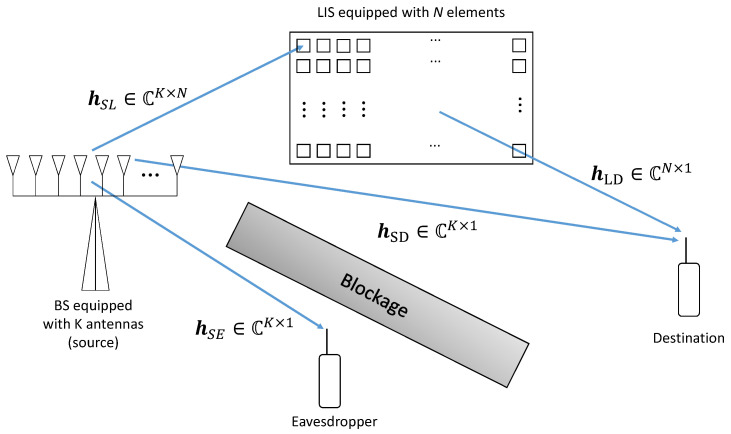
System model with eavesdropper link.

As shown in Figure 1, this study considers a base station (BS) equipped with an antenna array of *K* antennas transmitting the same signal to a unique single antenna user, the destination. Additionally, a large intelligent surface system with *N* reflecting elements aids the system. Both channels BS to LIS and LIS to the user are modeled by the Nakagami-*m* distribution. There is a direct link between the user and the BS and between an eavesdropper and the BS whose channel is also Nakagami-*m* distributed. The signal that arrives at the destination antenna is given by

(1) $$r=(h_{SL}^{H}\Phi^{H}h_{LD}^{H}+h_{SD}^{H})\Psi +\eta ,$$

where $h_{SL}\in C^{N\times 1}$ is the link between the source and the LIS, $h_{LD}\in C^{K\times N}$ is the link between the LIS and the destination and $h_{SD}\in C^{K\times 1}$ is the direct link between the source and the destination. The term $\Phi \in C^{N\times N}$ is a diagonal matrix, whose elements are the phase shifts $e^{-j\phi_{1}}\ldots e^{-j\phi_{N}}$ applied by the LIS to the incident electromagnetic waves. The LIS’s phases, $\phi_{n}\;\forall n$, are assumed continuous in the interval of 0 to 2π radians. The term Ψ=vs represents the precoded signal, where the data symbol is s∼CN(0,1) and the optimal precoding vector is applied by BS, according to the MRT (maximum ratio transmission) criterion, i.e.,

(2) $$v=\frac{h^{H}}{∥h∥}.$$

Finally, the term η∼CN(0,1) is additive white Gaussian noise (AWGN) with zero mean and unit variance. Suppose that there is no LoS in the direct link and that it is modeled as a complex normal random variable, with zero mean and variance $\sigma_{SD}^{2}$. Additionally, the magnitude of the channels $h_{i}=\left|h_{i}\right|e^{j\phi_{i}}$ with i∈{SL,LD} are Nakagami-*m* distributed with probability density function (PDF) given by

(3) $$f_{X}\left(x\right)=\frac{2m_{i}^{m_{i}}}{\Gamma \left(m_{i}\right)\Omega_{i}^{m_{i}}}x^{2m_{i}-1}e^{-\frac{m_{i}}{\Omega_{i}}x^{2}}.$$

In this work, the parameters $m_{i}$ and $\Omega_{i}$ refer to the shape and spread of the Nakagami-*m* PDF, respectively. The distribution of the phases is not specified since, for this model, these phases are not relevant. Then, the overall channel, including the LIS and the antenna array, can be defined as

(4) $$h=h_{SL}^{H}\Phi^{H}h_{LD}^{H}+h_{SD}^{H},$$

whose representation in scalar form is

(5) $$h_{k}=\sum_{i=1}^{N}\left|h_{ki}^{LD}\right|\left|h_{i}^{SL}\right|e^{j(\phi_{i}-\phi_{i}^{SL}-\phi_{ki}^{LD})}+{h_{k}}^{SD}.\;k\in N$$

Perfect phase cancelling occurs when $\phi_{i}-\phi_{i}^{SL}-\phi_{ki}^{LD}=0$. Therefore, in this scenario, it follows that $\phi_{i}=\phi_{i}^{SL}+\phi_{ki}^{LD}$. However, the task of removing the overall channel phase is unfeasible. Some residual phase noise is left behind, in this case, $\phi_{i}-\phi_{i}^{SL}-\phi_{ki}^{LD}=\theta_{ki}$, where $\theta_{ki}$ is the phase noise, which, in this work, is modeled as a Von Mises random variable with concentration parameter κ. Therefore, the overall channel can be written as

(6) $$h_{k}=\sum_{i=1}^{N}\left|h_{ki}^{LD}\right|\left|h_{i}^{SL}\right|e^{j\theta_{ki}}+h_{k}^{SD}.$$

It is expected that there is no phase error in the best case analysis, but this situation is entirely unfeasible. However, it is possible to estimate an optimal phase adjustment matrix that provides a performance as good as possible, so it is expected that, on average, the phase errors are zero. The zero mean Von Mises circular distribution can be proper to model the phases of each antenna’s fading coefficients [20]. It has nonzero support in the interval −π and π and a concentration parameter κ associated with the quality of the phase adjustment promoted by the LIS and the efficiency of the channel estimation method.

The moment-generating function (MGF) of the Von Mises distribution is useful since a complex exponential represents the phase adjustments. With the MGF, it is possible to calculate the statistical moments associated with the channel coefficients.

Let *X* be a Von Mises random variable; therefore, its MGF is given by $\varphi_{p}=E\left[e^{-jpX}\right]=\alpha_{p}+j\beta_{p}$. Since the zero mean Von Mises distribution is symmetric about zero, then the imaginary part of the MGF $\beta_{p}=E\left[\sin pX\right]=0$, and the real part is $\alpha_{p}=\frac{I_{p}\left(\kappa \right)}{I_{0}\left(\kappa \right)}$, where $I_{p}\left(\kappa \right)$ is the modified Bessel function of first kind and order *p*.

Considering that the precoder is the normalized hermitian of the overall channel, the SNR of the desired link is

(7) $$\gamma_{D}=\left(h_{SL}^{H}\Phi^{H}h_{LD}^{H}+h_{SD}^{H}\right)v={∥h∥}^{2}.$$

Assuming, as an approximation that $\gamma_{D}$ is Gamma distributed, then its statistical moments, α and β can be estimated as

(8) $$\alpha =\frac{E{\left[\gamma_{D}\right]}^{2}}{\mathrm{var}(\gamma_{D})},\;\;\beta =\frac{E[\gamma_{D}]}{\mathrm{var}(\gamma_{D})},$$

where α and β are the shape and rate parameters, while $E[\gamma_{D}]$ and $\mathrm{var}(\gamma_{D})$ are the expected value and variance of $\gamma_{D}$, respectively, as shown throughout Appendix A.

The assumption that the distribution of $\gamma_{D}$ is Gamma distributed can be assessed using the Hellinger distance. According to Beran [21], the Hellinger distance between two arbitrary discrete probability distributions $p_{k}$ and $q_{k}$ can be obtained as

(9) $$D_{HL}=\frac{1}{\sqrt{2}}\sqrt{\sum_{k=0}^{N_{p}-1}{\left(\sqrt{p_{k}}-\sqrt{q_{k}}\right)}^{2}},$$

where $N_{p}$ is the number of samples available to calculate the distance. The Hellinger distance is limited in the interval $0\leq D_{HL}\leq 1$ and can be considered as an absolute metric.

**Figure 2:**
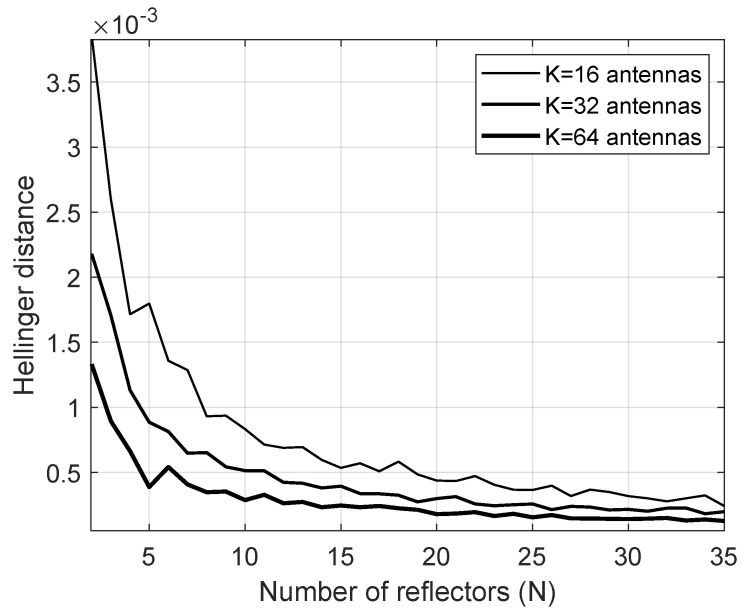
Hellinger distance.

In Figure 2, the realizations of a Monte Carlo simulation of the channels involved in the system are used to compose a histogram that approximates the PDF of the overall channel that is compared to the Gamma distribution predicted by the approximation proposed in this study. To perform the analysis, $10^{6}$ iterations were performed with unit variance for all channels, Von Mises concentration parameter κ=2 and the Nakagami parameter m=2. The results show that the Hellinger distance decreases when *N* and *K* increase. In the last case, for K=16, the decrease is even more pronounced. Therefore, this accurate approximation motivates us to formulate the problem further.

## 3. Problem Formulation

Knowing that the SNR can be approximated by a Gamma random variable, closed-form expressions for spectral efficiency, BER and SOP are derived in the following subsections.

### 3.1. Spectral Efficiency

The average spectral efficiency of the system can be defined as

(10) $$\begin{array}{c} C=E\left[\log_{2}\left(1+\gamma \right)\right]=\int_{0}^{\infty }\log_{2}\left(1+\gamma \right)\frac{\beta^{\alpha }}{\Gamma (\alpha )}\gamma^{\alpha -1}e^{-\beta \gamma }d\gamma , \end{array}$$

whose approximated solution is given by

(11) $$\begin{array}{c} C=\frac{{\left(-1\right)}^{-\alpha }\beta }{\log(2)\Gamma (\alpha )}\left[{\left(-1\right)}^{\alpha }\left(\Gamma \left(\alpha -1\right){\;}_{2}F_{2}\left(1,1;2,2-\alpha ;\beta \right)+\frac{\Gamma \left(\alpha \right)(\psi^{\left(0\right)}\left(\alpha \right)-\log\left(\beta \right))}{\beta }\right)+\pi \beta \csc\left(\pi \alpha \right)\left(\Gamma \left(\alpha \right)-\Gamma \left(\alpha ,-\beta \right)\right)\right], \end{array}$$

where $\psi^{\left(0\right)}\left(.\right)$ is the digamma function, Γ(.) is the gamma function, Γ(.,.) is the incomplete gamma function, and ${}_{2}F_{2}\left(a,b;c,d;e\right)$ is the generalized hypergeometric function. It is noteworthy that, although this study did not find an explicit solution for the spectral efficiency, it does present a more generic solution for the integral in the reference [22].

### 3.2. Bit Error Probability

The error probability for the *M*-QAM modulation can be approximately obtained by [23]

(12) $$P_{e}^{QAM}\left(\gamma \right)=1-{\left(1-2\left(1-\frac{1}{\sqrt{M}}\right)Q\left[\sqrt{\frac{3\gamma {log}_{2}M}{\left(M-1\right)}}\right]\right)}^{2}.$$

Assuming, as an approximation that γ is Gamma distributed, the mean bit error probability ${\bar{P}}_{e}^{QAM}$ can be calculated by

(13) $${\bar{P}}_{e}^{QAM}\left(\gamma \right)=\int_{0}^{\infty }P_{e}^{QAM}\left(\gamma v\right)f_{{∥w∥}^{2}}\left(v\right)dv,$$

where $f_{{∥w∥}^{2}}\left(v\right)$ is the pdf of ${∥w∥}^{2}$ as a function of an independent variable *v*.

Ferreira et al. [19] derived a close upper bound for the mean error probability of an *M*-QAM schema under Gamma fading by using the approximation

(14) $${\bar{P}}_{e}^{QAM}\left(\gamma \right)\approx \frac{4}{\log_{2}M}Q\left(\sqrt{\frac{3\gamma {log}_{2}M}{M-1}}\right).$$

From the Chernoff bound $Q\left(x\right)\leq \frac{1}{2}e^{-\frac{1}{2}x^{2}}$, they obtained the following upper bound for BER

(15) $${\bar{P}}_{e}^{QAM}\left(\gamma \right)<\frac{1.38629{\left(\frac{2.16404\gamma \log(M)}{(M-1)\beta }+1\right)}^{-\alpha }}{\log(M)},$$

which is close to the exact solution.

### 3.3. Secrecy Outage Probability

Considering that an eavesdropper has access to the signal provided by the source and according to [24], the secrecy capacity associated with the two fading channels can be obtained as

(16) $$C=\left\{\begin{array}{cc} \ln\left(1+\gamma_{D}\right)-\ln\left(1+\gamma_{E}\right) & \gamma_{D}>\gamma_{E}\\ 0 & \gamma_{D}\leq \gamma_{E} \end{array}\right.,$$

where $\gamma_{E}$ is the SNR of the link between the source and the eavesdropper. Therefore, the SOP is defined as the probability that the instantaneous secrecy capacity, *C*, be less than or equal to a given capacity threshold, $\ln\left(1+\gamma_{th}\right)$, which is expressed as

(17) $$\mathrm{SOP}=Pr\left[\ln\frac{\left(1+\gamma_{D}\right)}{\left(1+\gamma_{E}\right)}\leq \ln\left(1+\gamma_{th}\right)\right]=\int_{0}^{\infty }\int_{0}^{\left(1+\gamma_{E}\right)\left(1+\gamma_{D}\right)-1}f_{\gamma_{E}}\left(w\right)f_{\gamma_{D}}\left(u\right)dudw,$$

where Pr[.] denotes the probability of a random event.

Considering a Nakagami-*m* distributed eavesdropper channel, the SOP can be obtained as follows:

(18) $$\mathrm{SOP}=\int_{0}^{\infty }\int_{0}^{\left(\gamma_{th}+1\right)\left(x+1\right)-1}\frac{\beta^{\alpha }\left(2m^{m}x^{2m-1}\right)y^{\alpha -1}\exp\left(-\beta y\right)\exp\left(-\frac{mx^{2}}{\Omega }\right)}{\Gamma \left(\alpha \right)(\Omega^{m}\Gamma \left(m\right))}dydx$$

Solving the first integral, the remaining expression becomes

(19) $$\mathrm{SOP}=\int_{0}^{\infty }\frac{2e^{-\frac{mx^{2}}{\Omega }}m^{m}x^{2m-1}\Omega^{-m}\left[\Gamma \left(\alpha \right)-\Gamma \left(\alpha ,\beta \left(x+\gamma_{th}\left(1+x\right)\right)\right)\right]}{\Gamma (m)\Gamma (\alpha )}dx,$$

where the term $\Gamma \left(\alpha \right)-\Gamma (\alpha ,\beta \left(x+\gamma_{th}\left(1+x\right)\right))$ can be rewritten as function of the lower incomplete gamma function

(20) $$\gamma \left(s,x\right)≜\int_{0}^{x}t^{s-1}e^{-t}dt,$$

considering that Γ(s)=γ(s,x)+Γ(s,x).

Representing the exponential in terms of power series, it follows that the incomplete Gamma function can be written as

(21) $$\int_{0}^{x}t^{s-1}e^{-t}dt=\int_{0}^{x}\sum_{k=0}^{\infty }{\left(-1\right)}^{k}\frac{t^{s+k-1}}{k!}dt=x^{s}\sum_{k=0}^{\infty }\frac{{\left(-x\right)}^{k}}{k!(s+k)}.$$

Applying the expansion (21) in (19), and using the result obtained by the reference [22] in its table of integrals for integrands of type $x^{a}e^{-px^{2}}\gamma \left(\nu ,cx\right)$, the SOP can be rewritten as (22), where ${}_{p}{\tilde{F}}_{q}$ is the regularized ${}_{p}F_{q}$ hypergeometric function and $v=\frac{1+\gamma_{th}}{\gamma_{th}}$. Although this expression is an infinite sum, it is possible to verify, in Section 4, that the error is small if only the first term is considered to compute the SOP:

(22) $$\begin{array}{c} \mathrm{SOP}=\sum_{k=0}^{\infty }\frac{{\left(-1\right)}^{k}\beta^{\alpha +k}\gamma_{th}^{\alpha +k}}{\Gamma (\alpha )\Gamma (k+1)}\\ \times (\frac{\pi m^{m}2^{-\alpha -k}v^{-2m}\Omega^{-m}\Gamma \left(m+\frac{1}{2}\right)\csc\left(\pi \left(\alpha +k+2m\right)\right){\;}_{2}{\tilde{F}}_{2}\left(m,m+\frac{1}{2};\frac{1}{2}\left(k+2m+\alpha +1\right),\frac{1}{2}\left(k+2m+\alpha +2\right);-\frac{m}{v^{2}\Omega }\right)}{\Gamma (-k-\alpha +1)}\\ +\frac{\pi^{3/2}\Omega^{\frac{\alpha +k}{2}}m^{\frac{1}{2}\left(-\alpha -k\right)}v^{\alpha +k}}{2\Gamma (m)}(\frac{2\csc\left(\frac{1}{2}\pi \left(\alpha +k+2m\right)\right){\;}_{2}{\tilde{F}}_{2}\left(\frac{1}{2}\left(-k-\alpha \right),\frac{1}{2}\left(-k-\alpha +1\right);\frac{1}{2},\frac{1}{2}\left(-k-2m-\alpha +2\right);-\frac{m}{v^{2}\Omega }\right)}{\alpha +k}\\ -\frac{\sqrt{m}\sec\left(\frac{1}{2}\pi \left(\alpha +k+2m\right)\right){\;}_{2}{\tilde{F}}_{2}\left(\frac{1}{2}\left(-k-\alpha +1\right),\frac{1}{2}\left(-k-\alpha +2\right);\frac{3}{2},\frac{1}{2}\left(-k-2m-\alpha +3\right);-\frac{m}{v^{2}\Omega }\right)}{v\sqrt{\Omega }})), \end{array}$$

## 4. Numerical Results

This section analyzes the accuracy of the proposed approximations and discusses the improvements in capacity provided by LIS. In unspecified cases, this study adopts, by default, the Nakagami-*m* shape parameters $m_{SL}=m_{LD}=m=2$. The spread parameters $\Omega_{SL}=\Omega_{LD}=\Omega$ were chosen to make the variances $\sigma_{SL}^{2}=\sigma_{LD}^{2}=1$, the Von Mises concentration parameter κ=2, K=16 antennas at the source, the size of the *M*-QAM constellation is M=16, and the number of iterations is $10^{6}$ for each Monte Carlo simulation.

For each iteration of the Monte Carlo method, we generate the coefficients $h_{k}$ of (6), the magnitudes are generated using the Nakagami-*m* distribution and the phase errors with the Von Mises distribution. Given the coefficients, it is easy to estimate the bit error rate, spectral efficiency, and the SOP in each realization of the random variables and approximating the simulated results by the mean value of these quantities. We compare each of the simulated results in several iterations with the theoretical formulas described in terms of channel parameters.

**Figure 3:**
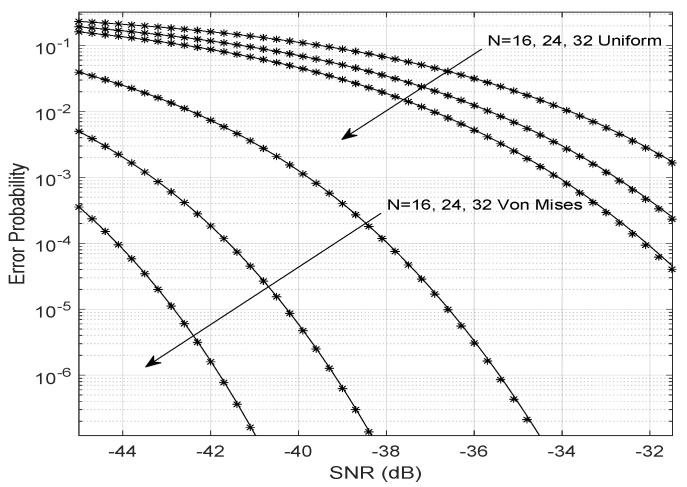
Bit error probability varying the SNR.

Figure 3 shows the simulated and theoretical BERs considering the Von Mises and uniformly distributed phase errors. The theoretical BER is obtained assuming that the overall fading channel has a Gamma distribution. Note that the larger the number of reflectors, *N*, the smaller the error probability for any SNR value. When the phase errors are uniformly distributed (κ=0), the error probability is higher than in the Von Mises scenario. This result shows the importance of accurately estimating the phases and channel gains and choosing the optimization method to find the best LIS phase shifts. Uniformly distributed phase noise indicates that the algorithm has equal chances to present significant phase errors (close to ±π) or small phase errors (close to zero). That implies greater bit error probabilities, which can be compensated only with a large number of antennas at the transmitter or with a large number of reflectors at the LIS.

**Figure 4:**
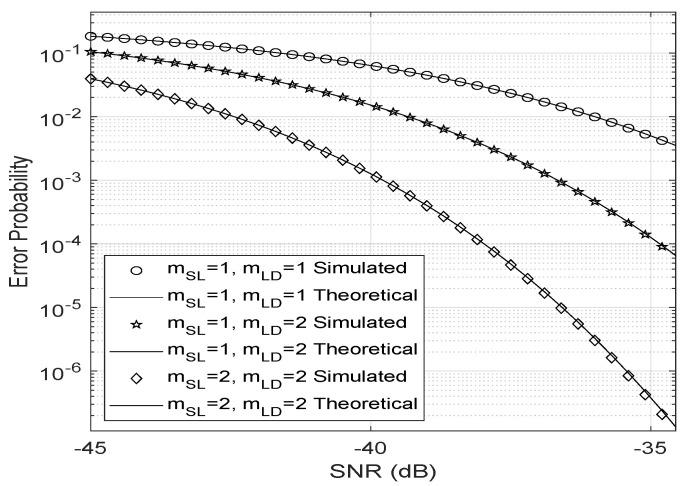
Bit error probability varying the LoS strength.

In its turn, Figure 4 confirms that large reflecting surfaces can produce an LoS link between the transmitter and the user even in a far-field Rayleigh fading channel. However, in a near-field scenario, a stronger LoS link (higher Nakagami-m parameter) implies a lower probability of error.

Even in weak LoS scenarios, LIS can decrease the probability of bit error by creating an LoS that is the result of beamforming toward the target user. Figure 3 shows that, for an environment with a fixed value of *m*, the increase in the number of reflectors (*N*), or the improvement of the phase adjustment performed by the LIS (related to the concentration parameter κ) can reduce the bit error rate in an aided LIS system.

**Figure 5:**
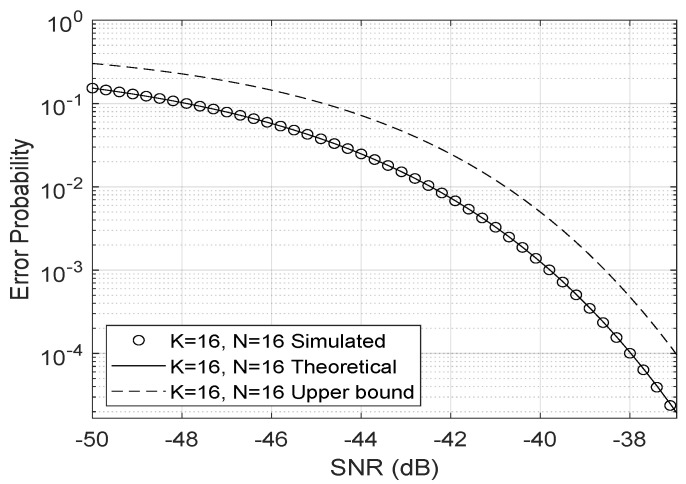
Bit error probability upper bound.

The upper bound (15) for the error probability proposed by Ferreira et al. [19] is very close to the bit error rate as shown in Figure 5, even when the fading coefficients are Nakagami-*m* distributed.

**Figure 6:**
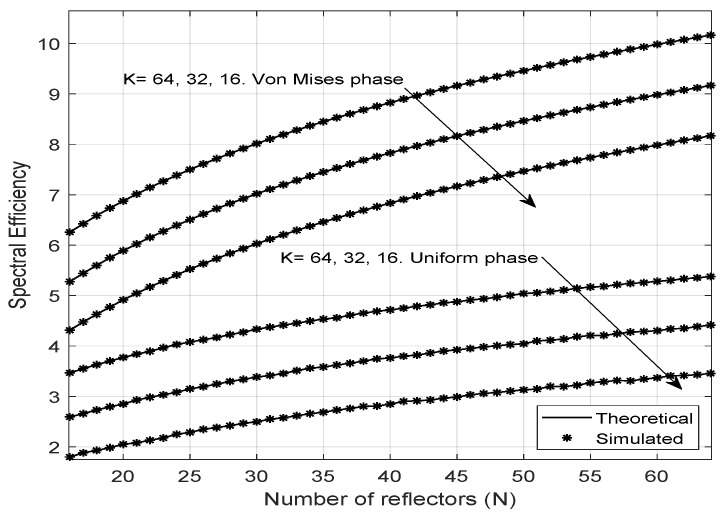
Spectral Efficiency varying *M* and *N*.

Regarding spectral efficiency, this study considers two scenarios. The first one has uniformly distributed phase noise, and the second one has a Von Mises distributed phase, as shown in Figure 6. Notably, the spectral efficiency increases when the LIS has a more significant number of reflectors, thus indicating a better sharing of the spectrum for the transmission of signals for a multiuser scenario. Moreover, the efficiency is higher for the case in which the phase errors have a Von Mises distribution and lower when the phase errors are uniformly distributed, which means that the phase adjustment of the LIS is a highly relevant factor in improving the spectral efficiency, reinforcing the importance of channel estimation and choice of the phase correction applied to reflectors. When the phase error distribution is more concentrated around zero (higher κ values), then the spectral efficiency is higher. Using an array of antennas on the base station can be a good choice to achieve better spectrum sharing in diverse scenarios. It is also remarkable that the result predicted by the formula proposed for the spectral efficiency is very close to the results obtained by the Monte Carlo simulation.

**Figure 7:**
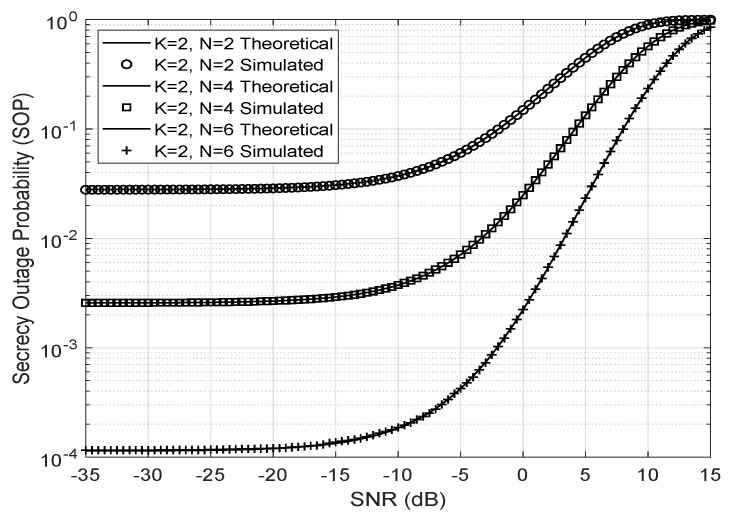
SOP for K=2 antennas and different number of reflectors (*N*).

In Figure 7, the secrecy outage probability for a Nakagami-m eavesdropper link with Ω=1 and m=1.4 is shown. The sum was truncated up to the index 1000, and the number of iterations used was $10^{6}$ to generate the Gamma distributed random SNR with parameters α and β, the Von Mises concentration parameter κ=2, K=2 antennas, unity variance, and Nakagami-*m* fading distribution for all channels between the antennas, the LIS, and the user. The larger the number of reflectors or the SNR, then the greater is the SOP.

**Figure 8:**
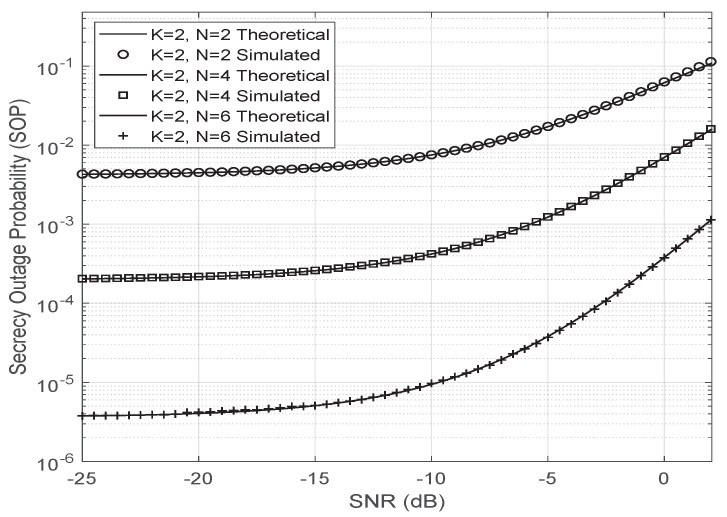
First order approximation of SOP for K=2 antennas and different number of reflectors (*N*).

The first-order approximation of the SOP, considering that the Nakagami-*m* parameters are m=2.5 and Ω=0.1 for all the channels in the system model, is also close to the simulated result as shown in Figure 8.

## 5. Final Considerations

This work has presented an in-depth analysis of the performance of systems aided by large intelligent surfaces considering the existence of an eavesdropper link in generic scenarios that contemplate channels with and without LoS links, employing the Nakagami-*m* distribution, and channels with or without a direct link to the transmitter and the user. This study derives very accurate analytical expressions from computing the secrecy outage probability, bit error probability, and secrecy capacity, in addition to reasonable approximations for estimating the equivalent channel parameters based on the central limit theorem.

## Appendix A. Parameters of *γ_D_*

It is possible to obtain the parameters of the $\gamma_{D}$ distribution, following the steps below.

### Appendix A.1. Expected Value of Each Fading Coefficient

Since the expected value is a linear operator, then

(A1) $$E\left[h_{k}\right]=E[\sum_{i=1}^{N}|h_{ki}^{LD}\left|\left|h_{i}^{SL}\right|e^{j\theta_{ki}}+h_{k}^{SD}\right]=NE\left[\left|h_{ki}^{LD}h_{i}^{SL}\right|e^{j\theta_{ki}}\right]+E\left[h_{k}^{SD}\right].$$

Since the channels are independent and identically distributed, $E[h_{k}^{SD}]=0$, and $E\left[e^{j\theta_{ki}}\right]=\alpha_{1}$, thus

(A2) $$E\left[h_{k}\right]=N\;\mu_{LD}\times \mu_{SL}\times \alpha_{1},$$

where

(A3) $$\mu_{LD}=E\left[\left|h_{ki}^{LD}\right|\right]=\frac{\Gamma \left(m_{LD}+0.5\right)}{\Gamma \left(m_{LD}\right)}{\left(\frac{\Omega_{LD}}{m_{LD}}\right)}^{\frac{1}{2}},$$

and

(A4) $$\mu_{SL}=E\left[\left|h_{i}^{SL}\right|\right]=\frac{\Gamma \left(m_{SL}+0.5\right)}{\Gamma \left(m_{SL}\right)}{\left(\frac{\Omega_{SL}}{m_{SL}}\right)}^{\frac{1}{2}},$$

are the expected values of each Nakagami-*m* channel. To obtain the variance of the overall channel fading coefficient, the mean of $c_{k}=Re\left\{h_{k}\right\}$ and $s_{k}=Im\left\{h_{k}\right\}$ needs to be calculated, i.e., the in-phase and quadrature components of the fading coefficient, respectively. The in-phase component can be written as

(A5) $$c_{k}=\sum_{i=1}^{N}\left|h_{ki}^{LD}\right|\left|h_{i}^{SL}\right|\cos\theta_{ki}+Re\left\{h_{k}^{SD}\right\},$$

while the quadrature component is

(A6) $$s_{k}=\sum_{i=1}^{N}\left|h_{ki}^{LD}\right|\left|h_{i}^{SL}\right|\sin\theta_{ki}+Im\left\{h_{k}^{SD}\right\}.$$

Then, the expected value of $c_{k}$ is

(A7) $$E\left[c_{k}\right]=\sum_{i=1}^{N}E\left[\left|h_{ki}^{LD}\right|\left|h_{i}^{SL}\right|\cos\theta_{ki}\right]+E\left[Re\left\{h_{k}^{SD}\right\}\right].$$

Since $E[Re\left\{h_{k}^{SD}\right\}]=0$ and all the summation terms are independent,

(A8) $$E\left[c_{k}\right]=N\times E\left[\left|h_{ki}^{LD}\right|\right]E\left[\left|h_{i}^{SL}\right|\right]E\left[\cos\theta_{ki}\right]N\times \mu_{LD}\;\mu_{SL}\;\alpha_{1},$$

as well as $E[h_{k}]$.

In its turn, the expected value of $s_{k}$ is given by

(A9) $$E\left[s_{k}\right]=\sum_{i=1}^{N}E\left[\left|h_{ki}^{LD}\right|\left|h_{i}^{SL}\right|\sin\theta_{ki}\right]+E\left[Im\left\{h_{k}^{SD}\right\}\right].$$

Since $E[Im\left\{h_{k}^{SD}\right\}]=0$ and all the summation terms are independent,

(A10) $$E\left[s_{k}\right]=N\times E\left[\left|h_{ki}^{LD}\right|\right]E\left[\left|h_{i}^{SL}\right|\right]E\left[\sin\theta_{ki}\right]=0,$$

since $E[\sin\theta_{ki}]=0$.

### Appendix A.2. Variance of the In-Phase and Quadrature Components of Each Fading Coefficient

The variance of the in-phase component is written as

(A11) $$\mathrm{var}\left(c_{k}\right)=\mathrm{var}\left(\sum_{i=1}^{N}\left|h_{ki}^{LD}\right|\left|h_{i}^{SL}\right|\cos\theta_{ki}+Re\left\{h_{k}^{SD}\right\}\right).$$

Since $\mathrm{var}\left(h_{k}^{SD}\right)=\sigma_{SD}^{2}$, the summation terms and $Re\{h_{k}^{SD}\}$ are independent and the $h_{k}^{SD}$ coefficient is zero mean, then

(A12) $$\mathrm{var}\left(c_{k}\right)=N\times \mathrm{var}\left(\left|h_{ki}^{LD}\right|\left|h_{i}^{SL}\right|\cos\theta_{ki}\right)+\frac{\sigma_{SD}^{2}}{2}.$$

Next, the variance of the term $\left|h_{ki}^{LD}\right|\left|h_{i}^{SL}\right|\cos\theta_{ki}$ is needed, considering that the variance of the product of two random variables *X* and *Y* is $\mathrm{var}\left(XY\right)=\mathrm{var}\left(X\right)\mathrm{var}\left(Y\right)+\mathrm{var}\left(X\right)E{\left[Y\right]}^{2}+\mathrm{var}\left(Y\right)E{\left[X\right]}^{2}$ and the phase noise is independent of the fading magnitudes. Thus,

(A13) $$\mathrm{var}\left(\left|h_{ki}^{LD}\right|\left|h_{i}^{SL}\right|\cos\theta_{ki}\right)=\mathrm{var}\left(\left|h_{ki}^{LD}\right|\left|h_{i}^{SL}\right|\right)\mathrm{var}\left(\cos\theta_{ki}\right)+\ldots \mathrm{var}\left(\left|h_{ki}^{LD}\right|\left|h_{i}^{SL}\right|\right){\left(E\left[\cos\theta_{ki}\right]\right)}^{2}+\mathrm{var}\left(\cos\theta_{ki}\right){\left(E\left[\left|h_{ki}^{LD}\right|\left|h_{i}^{SL}\right|\right]\right)}^{2}.$$

Since $\left|h_{ki}^{LD}\right|$ and $\left|h_{i}^{SL}\right|$ are independents,

(A14) $$\mathrm{var}\left(\left|h_{ki}^{LD}\right|\left|h_{i}^{SL}\right|\right)=\mathrm{var}\left(\left|h_{ki}^{LD}\right|\right)\mathrm{var}\left(\left|h_{i}^{SL}\right|\right)+\mathrm{var}\left(\left|h_{ki}^{LD}\right|\right){\left(E\left[\left|h_{i}^{SL}\right|\right]\right)}^{2}+\ldots \mathrm{var}\left(\left|h_{i}^{SL}\right|\right){\left(E\left[\left|h_{ki}^{LD}\right|\right]\right)}^{2}$$

and

(A15) $$E\left[\left|h_{ki}^{LD}\right|\left|h_{i}^{SL}\right|\right]=\mu_{LD}\times \mu_{SL}.$$

Next, considering that $\mathrm{var}\left(\left|h_{i}^{SL}\right|\right)=\sigma_{SL}^{2}$ and $\mathrm{var}\left(\left|h_{ki}^{LD}\right|\right)=\sigma_{LD}^{2}$, then

(A16) $$\mathrm{var}\left(\left|h_{ki}^{LD}\right|\left|h_{i}^{SL}\right|\right)=\sigma_{LD}^{2}\sigma_{SL}^{2}+\sigma_{LD}^{2}\mu_{SL}^{2}+\sigma_{SL}^{2}\mu_{LD}^{2}.$$

By using (A16), (A13) can thus be evaluated as

(A17) $$\mathrm{var}\left(\left|h_{ki}^{LD}\right|\left|h_{i}^{SL}\right|\cos\theta_{ki}\right)=\left(\sigma_{LD}^{2}\sigma_{SL}^{2}+\sigma_{LD}^{2}\mu_{SL}^{2}+\sigma_{SL}^{2}\mu_{LD}^{2}\right)\times \frac{1}{2}\left(1+\alpha_{2}\right)+\left(\sigma_{LD}^{2}\sigma_{SL}^{2}+\sigma_{LD}^{2}\mu_{SL}^{2}+\sigma_{SL}^{2}\mu_{LD}^{2}\right)\alpha_{1}^{2}+\frac{1}{2}\left(1+\alpha_{2}\right)\times \mu_{SL}^{2}\mu_{LD}^{2},$$

which can be rewritten as

(A18) $$\mathrm{var}\left(\left|h_{ki}^{LD}\right|\left|h_{i}^{SL}\right|\cos\theta_{ki}\right)=\frac{1}{2}\left(1+\alpha_{2}\right)\times \mu_{SL}^{2}\mu_{LD}^{2}+\ldots \frac{1}{2}\left(\sigma_{LD}^{2}\sigma_{SL}^{2}+\sigma_{LD}^{2}\mu_{SL}^{2}+\sigma_{SL}^{2}\mu_{LD}^{2}\right)\left(1+\alpha_{2}+2\alpha_{1}^{2}\right).$$

Therefore, the variance of the in-phase component is

(A19) $$\mathrm{var}\left(c_{k}\right)=\frac{\sigma_{SD}^{2}}{2}+\frac{N}{2}\times \left[\left(1+\alpha_{2}\right)\times \mu_{SL}^{2}\mu_{LD}^{2}+\ldots \left(\sigma_{LD}^{2}\sigma_{SL}^{2}+\sigma_{LD}^{2}\mu_{SL}^{2}+\sigma_{SL}^{2}\mu_{LD}^{2}\right)\left(1+\alpha_{2}+2\alpha_{1}^{2}\right)\right].$$

On other hand, the variance of the quadrature component of each fading coefficient is given by

(A20) $$\mathrm{var}\left(s_{k}\right)=\mathrm{var}\left(\sum_{i=1}^{N}\left|h_{ki}^{LD}\right|\left|h_{i}^{SL}\right|\sin\theta_{ki}+Im\left\{h_{k}^{SD}\right\}\right).$$

Considering that $\mathrm{var}\left(h_{k}^{SD}\right)=\sigma_{SD}^{2}$, the summation terms and $Im\{h_{k}^{SD}\}$ are independent and the term $h_{k}^{SD}$ is zero mean, then

(A21) $$\mathrm{var}\left(s_{k}\right)=N\times \mathrm{var}\left(\left|h_{ki}^{LD}\right|\left|h_{i}^{SL}\right|\sin\theta_{ki}\right)+\frac{\sigma_{SD}^{2}}{2}.$$

Next, for that reason,

(A22) $$\mathrm{var}\left(\left|h_{ki}^{LD}\right|\left|h_{i}^{SL}\right|\sin\theta_{ki}\right)=\mathrm{var}\left(\left|h_{ki}^{LD}\right|\left|h_{i}^{SL}\right|\right)\mathrm{var}\left(\sin\theta_{ki}\right)+\ldots \mathrm{var}\left(\left|h_{ki}^{LD}\right|\left|h_{i}^{SL}\right|\right){\left(E\left[\sin\theta_{ki}\right]\right)}^{2}+\mathrm{var}\left(\sin\theta_{ki}\right){\left(E\left[\left|h_{ki}^{LD}\right|\left|h_{i}^{SL}\right|\right]\right)}^{2}.$$

Since $E[\sin\theta_{ki}]=0$, applying (A16) therefore

(A23) $$\mathrm{var}\left(\left|h_{ki}^{LD}\right|\left|h_{i}^{SL}\right|\sin\theta_{ki}\right)=\frac{1}{2}\left(1-\alpha_{2}\right)\mu_{SL}^{2}\mu_{LD}^{2}+\ldots \frac{1}{2}\left(1-\alpha_{2}\right)\left(\sigma_{LD}^{2}\sigma_{SL}^{2}+\sigma_{LD}^{2}\mu_{SL}^{2}+\sigma_{SL}^{2}\mu_{LD}^{2}\right),$$

which can be rewritten as

(A24) $$\mathrm{var}\left(\left|h_{ki}^{LD}\right|\left|h_{i}^{SL}\right|\sin\theta_{ki}\right)=\frac{1}{2}\left(1-\alpha_{2}\right)\left(\sigma_{LD}^{2}\sigma_{SL}^{2}+\sigma_{LD}^{2}\mu_{SL}^{2}+\sigma_{SL}^{2}\mu_{LD}^{2}+\mu_{SL}^{2}\mu_{LD}^{2}\right).$$

Therefore, the variance of the quadrature component is expressed by

(A25) $$\mathrm{var}\left(s_{k}\right)=\frac{\sigma_{SD}^{2}}{2}+\frac{N\left(1-\alpha_{2}\right)\left(\sigma_{LD}^{2}\sigma_{SL}^{2}+\sigma_{LD}^{2}\mu_{SL}^{2}+\sigma_{SL}^{2}\mu_{LD}^{2}+\mu_{SL}^{2}\mu_{LD}^{2}\right)}{2}.$$

### Appendix A.3. Expected Value of γ_D_

All the fading coefficients are independent and identically distributed and $E\left[\gamma_{D}\right]=E\left[{∥h∥}^{2}\right]$. Therefore,

(A26) $$E\left[\gamma_{D}\right]=E\left[\sum_{k=1}^{K}{\left|h_{k}\right|}^{2}\right]=K\times E\left[{\left|h_{k}\right|}^{2}\right].$$

Considering that $E\left[{\left|h_{k}\right|}^{2}\right]=E\left[c_{k}^{2}+s_{k}^{2}\right]=E\left[c_{k}^{2}\right]+E\left[s_{k}^{2}\right]$, then

$$E\left[c_{k}^{2}\right]=\mathrm{var}\left(c_{k}\right)+{\left(E\left[c_{k}\right]\right)}^{2},$$

and, by using (A19) and (A25),

(A27) $$E\left[c_{k}^{2}\right]=\frac{\sigma_{SD}^{2}}{2}+N^{2}\times \mu_{LD}^{2}\;\mu_{SL}^{2}\;\alpha_{1}^{2}+\frac{N}{2}\times \left[\left(1+\alpha_{2}\right)\times \mu_{SL}^{2}\mu_{LD}^{2}+\ldots \left(\sigma_{LD}^{2}\sigma_{SL}^{2}+\sigma_{LD}^{2}\mu_{SL}^{2}+\sigma_{SL}^{2}\mu_{LD}^{2}\right)\left(1+\alpha_{2}+2\alpha_{1}^{2}\right)\right].$$

For the quadrature component, consider that $E[s_{k}]=0$, and, consequently, $E\left[s_{k}^{2}\right]=\mathrm{var}\left(s_{k}\right)$. Then,

(A28) $$E\left[s_{k}^{2}\right]=\frac{\sigma_{SD}^{2}}{2}+\frac{N\left(1-\alpha_{2}\right)\left(\sigma_{LD}^{2}\sigma_{SL}^{2}+\sigma_{LD}^{2}\mu_{SL}^{2}+\sigma_{SL}^{2}\mu_{LD}^{2}+\mu_{SL}^{2}\mu_{LD}^{2}\right)}{2}.$$

and the mean value of the overall fading coefficient magnitude is given by

(A29) $$\begin{array}{c} E\left[\gamma_{D}\right]=\frac{K}{2}\times (2\sigma_{SD}^{2}+2N^{2}\times \mu_{LD}^{2}\;\mu_{SL}^{2}\;\alpha_{1}^{2}N[\left(1+\alpha_{2}\right)\times \mu_{SL}^{2}\mu_{LD}^{2}+\ldots \\ \left(\sigma_{LD}^{2}\sigma_{SL}^{2}+\sigma_{LD}^{2}\mu_{SL}^{2}+\sigma_{SL}^{2}\mu_{LD}^{2}\right)\left(1+\alpha_{2}+2\alpha_{1}^{2}\right)]+\ldots \\ N\left(1-\alpha_{2}\right)\left(\sigma_{LD}^{2}\sigma_{SL}^{2}+\sigma_{LD}^{2}\mu_{SL}^{2}+\sigma_{SL}^{2}\mu_{LD}^{2}+\mu_{SL}^{2}\mu_{LD}^{2}\right)). \end{array}$$

### Appendix A.4. Variance of γ_D_

Let $Z_{i}={\left|h_{i}\right|}^{2}$; then,

(A30) $$\mathrm{var}\left(\gamma_{D}\right)=\mathrm{var}\left({∥h∥}^{2}\right)=\mathrm{var}\left(\sum_{i=1}^{M}Z_{i}\right).$$

Therefore, the variance of the sum of the terms $Z_{i}$ is given by

(A31) $$\mathrm{var}\left(\sum_{i=1}^{M}Z_{i}\right)=\sum_{i=1}^{M}\mathrm{var}\left(Z_{i}\right)+\;2\sum_{1\leq i<k\leq M}\mathrm{cov}\left(Z_{i},Z_{k}\right),$$

whose magnitudes are equally distributed. Therefore,

(A32) $$\mathrm{var}\left(\sum_{i=1}^{M}Z_{i}\right)=M\;\mathrm{var}\left(Z_{i}\right)+M\left(M-1\right)\;\;\mathrm{cov}\left(Z_{i},Z_{k}\right).$$

The covariance can be obtained by

(A33) $$\mathrm{cov}\left(Z_{i},Z_{k}\right)=E\left[Z_{i}Z_{k}\right]-E\left[Z_{i}\right]E\left[Z_{k}\right],$$

where

(A34) $$E\left[Z_{i}Z_{k}\right]=E\left[c_{i}^{2}c_{k}^{2}\right]+2E\left[c_{i}^{2}s_{k}^{2}\right]+E\left[s_{i}^{2}s_{k}^{2}\right].$$

The expected value of the product of two different in-phase coefficients can be written as

(A35) $$E\left[c_{i}c_{k}\right]=E\left[\left(\sum_{l=1}^{N}\left|h_{il}^{LD}\right|\left|h_{l}^{SL}\right|\cos\theta_{il}+Re\left\{h_{i}^{SD}\right\}\right)\times \left(\sum_{m=1}^{N}\left|h_{km}^{LD}\right|\left|h_{m}^{SL}\right|\cos\theta_{km}+Re\left\{h_{k}^{SD}\right\}\right)\right].$$

By expanding the product,

(A36) $$E\left[c_{i}c_{k}\right]=E\left[Re\left\{h_{i}^{SD}\right\}Re\left\{h_{k}^{SD}\right\}\right]+\sum_{l=1}^{N}\sum_{m=1}^{N}E\left[\left|h_{il}^{LD}\right|\left|h_{km}^{LD}\right|\left|h_{l}^{SL}\right|\left|h_{m}^{SL}\right|\cos\theta_{il}\cos\theta_{km}\right],$$

where the independent terms can be separated as

(A37) $$E\left[\left|h_{il}^{LD}\right|\left|h_{km}^{LD}\right|\left|h_{l}^{SL}\right|\left|h_{m}^{SL}\right|\cos\theta_{il}\cos\theta_{km}\right]=E{\left[\left|h_{km}^{LD}\right|\right]}^{2}E{\left[\left|h_{m}^{SL}\right|\right]}^{2}E{\left[\cos\theta_{km}\right]}^{2}=\mu_{LD}^{2}\mu_{SL}^{2}\alpha_{1}^{2}\forall l\neq m,$$

and

(A38) $$E\left[\left|h_{il}^{LD}\right|\left|h_{km}^{LD}\right|\left|h_{l}^{SL}\right|\left|h_{m}^{SL}\right|\cos\theta_{il}\cos\theta_{km}\right]=E{\left[\left|h_{km}^{LD}\right|\right]}^{2}E\left[{\left|h_{m}^{SL}\right|}^{2}\right]E\left[\cos\theta_{im}\right]E\left[\cos\theta_{km}\right]=\mu_{LD}^{2}\left(\sigma_{SL}^{2}+\mu_{SL}^{2}\right)\alpha_{1}^{2}\forall i\neq k,l=m,$$

where the term $E\left[{\left|h_{m}^{SL}\right|}^{2}\right]=\sigma_{SL}^{2}+\mu_{SL}^{2}$ and the variance of the Nakagami-*m* distributed term is

(A39) $$\sigma_{SL}^{2}=\Omega_{SL}\left(1-\frac{1}{m_{SL}}{\left(\frac{\Gamma (m_{SL}+0.5)}{\Gamma (m_{SL})}\right)}^{2}\right).$$

For i=k,

(A40) $$E\left[\left|h_{il}^{LD}\right|\left|h_{km}^{LD}\right|\left|h_{l}^{SL}\right|\left|h_{m}^{SL}\right|\cos\theta_{il}\cos\theta_{km}\right]=E\left[{\left|h_{km}^{LD}\right|}^{2}\right]E\left[{\left|h_{m}^{SL}\right|}^{2}\right]E\left[\cos^{2}\theta_{km}\right]=\frac{1}{2}\left(\sigma_{LD}^{2}+\mu_{LD}^{2}\right)\left(\sigma_{SL}^{2}+\mu_{SL}^{2}\right)\left(1+\alpha_{2}\right)\forall l=m,i=k,$$

where

(A41) $$\sigma_{LD}^{2}=\Omega_{LD}\left(1-\frac{1}{m_{LD}}{\left(\frac{\Gamma (m_{LD}+0.5)}{\Gamma (m_{LD})}\right)}^{2}\right).$$

To obtain the variance according to (A34), the term $E[c_{i}^{2}c_{k}^{2}]$ needs to be computed. The terms are approximately correlated Gaussian random variables by the central limit theorem (CLT), for large values of *N*, therefore

(A42) $$E\left[c_{i}^{2}c_{k}^{2}\right]=\int_{-\infty }^{\infty }\int_{-\infty }^{\infty }x^{2}y^{2}f_{c_{i},c_{k}}\left(x,y\right)dxdy,$$

where $f_{c_{i},c_{k}}\left(x,y\right)$ is the joint distribution of the two correlated Gaussian variables $c_{i}$ and $c_{k}$. The result of the integral is

(A43) $$E\left[c_{i}^{2}c_{k}^{2}\right]=\mu_{c_{k}}^{4}+2\mu_{c_{k}}^{2}\left(1+2\rho_{c_{i},c_{k}}\right)\sigma_{c_{k}}^{2}+\left(1+2\rho_{c_{i},c_{k}}^{2}\right)\sigma_{c_{k}}^{4},$$

where $\mu_{c_{k}}=E\left[c_{k}\right]$, $\sigma_{c_{k}}^{2}=\mathrm{var}\left(c_{k}\right)$, and since $\mathrm{var}\left(c_{i}\right)=\mathrm{var}\left(c_{k}\right)$ and $E\left[c_{i}\right]=E\left[c_{k}\right]$, the correlation coefficient $\rho_{c_{i},c_{k}}$ can be obtained as

(A44) $$\rho_{c_{i},c_{k}}=\frac{E\left[c_{i}c_{k}\right]-\mu_{c_{k}}^{2}}{\mathrm{var}(c_{k})},$$

where $E[c_{i}c_{k}]$ can be calculated by (A45) in the next page.

(A45) $$E\left[c_{i}c_{k}\right]=\left\{\begin{array}{c} N\left(N-1\right)\left(\mu_{LD}^{2}\mu_{SL}^{2}\alpha_{1}^{2}\right)+N\left(\mu_{LD}^{2}\left(\sigma_{SL}^{2}+\mu_{SL}^{2}\right)\alpha_{1}^{2}\right)i\neq k\\ N\left(N-1\right)\left(\mu_{LD}^{2}\mu_{SL}^{2}\alpha_{1}^{2}\right)+\frac{N}{2}\left(\left(\sigma_{LD}^{2}+\mu_{LD}^{2}\right)\left(\sigma_{SL}^{2}+\mu_{SL}^{2}\right)\left(1+\alpha_{2}\right)\right)+\frac{1}{2}\sigma_{SD}^{2}i=k \end{array}\right.$$

Since the moments of $c_{k}$ were previously calculated, $\rho_{c_{i},c_{k}}$ can be obtained by

(A46) $$\rho_{c_{i},c_{k}}=\frac{N\left(N-1\right)\left(\mu_{LD}^{2}\mu_{SL}^{2}\alpha_{1}^{2}\right)+N\left(\mu_{LD}^{2}\left(\sigma_{SL}^{2}+\mu_{SL}^{2}\right)\alpha_{1}^{2}\right)-{\left(N\;\mu_{LD}\;\mu_{SL}\;\alpha_{1}\right)}^{2}}{\frac{\sigma_{SD}^{2}}{2}+\frac{N}{2}\left[\left(1+\alpha_{2}\right)\times \mu_{SL}^{2}\mu_{LD}^{2}+\left(\sigma_{LD}^{2}\sigma_{SL}^{2}+\sigma_{LD}^{2}\mu_{SL}^{2}+\sigma_{SL}^{2}\mu_{LD}^{2}\right)\left(1+\alpha_{2}+2\alpha_{1}^{2}\right)\right]}\;\;i\neq k.$$

In its turn, applying (A46) for the correlation coefficient $\rho_{c_{i},c_{k}}$ in the definition of $E[c_{i}^{2}c_{k}^{2}]$ in (A43), it is possible to find that (A47).

(A47) $$E\left[c_{i}^{2}c_{k}^{2}\right]=\left\{\begin{array}{ll} a_{1}^{4}\left(1-\frac{4}{N}\right)+a_{1}^{2}\left(\sigma_{SD}^{2}+a_{4}+Na_{2}a_{3}-\frac{2}{N}+4a_{5}\right)2a_{5}+\frac{1}{4}{\left(\sigma_{SD}^{2}+a_{4}+Na_{2}a_{3}\right)}^{2} & i\neq k\\ a_{1}^{4}+3a_{1}^{2}\left(\sigma_{SD}^{2}+a_{4}+Na_{2}a_{3}\right)+\frac{3}{4}\left(\sigma_{SD}^{2}+a_{4}+Na_{2}a_{3}\right) & i=k \end{array}\right.$$

where $a_{1}=N\mu_{SL}\mu_{LD}\alpha_{1},\;a_{2}=\sigma_{LD}^{2}\sigma_{SL}^{2}+\sigma_{LD}^{2}\mu_{SL}^{2}+\sigma_{SL}^{2}\mu_{LD}^{2},\;a_{3}=1+\alpha_{2}+2\alpha_{1}^{2},\;a_{4}=N\mu_{SL}^{2}\mu_{LD}^{2}\left(1+\alpha_{2}\right),\;\;\;\;\;\;\;\;\;\;\;$$a_{5}=N\mu_{LD}^{2}\alpha_{1}^{2}\left(\sigma_{SL}^{2}+\mu_{SL}^{2}\right)$. The term $E[c_{i}^{2}s_{k}^{2}]$ depends on two uncorrelated but not independent random variables $c_{i}$ and $s_{k}$, the correlation is zero and, to calculate the correlation of the squared product, it is necessary to expand the very definition of the two terms as shown in

(A48) $$E\left[c_{i}^{2}s_{k}^{2}\right]=E\left[{\left(\sum_{l=1}^{N}\left|h_{il}^{LD}\right|\left|h_{l}^{SL}\right|\cos\theta_{il}+Re\left\{h_{i}^{SD}\right\}\right)}^{2}\times {\left(\sum_{m=1}^{N}\left|h_{km}^{LD}\right|\left|h_{m}^{SL}\right|\sin\theta_{km}+Im\left\{h_{k}^{SD}\right\}\right)}^{2}\right]$$

Expanding the product in (A48), it follows that

(A49) $$\begin{array}{c} E[{\left(\sum_{l=1}^{N}\left|h_{il}^{LD}\right|\left|h_{l}^{SL}\right|\cos\theta_{il}\right)}^{2}{\left(\sum_{m=1}^{N}\left|h_{km}^{LD}\right|\left|h_{m}^{SL}\right|\sin\theta_{km}\right)}^{2}]=\\ E[\sum_{l=1}^{N}\sum_{t=1}^{N}\sum_{d=1}^{N}\sum_{m=1}^{N}\left|h_{il}^{LD}\right|\left|h_{it}^{LD}\right|\left|h_{kd}^{LD}\right|\left|h_{km}^{LD}\right|\left|h_{l}^{SL}\right|\left|h_{m}^{SL}\right|\left|h_{d}^{SL}\right|\left|h_{m}^{SL}\right|\cos\theta_{il}\cos\theta_{it}\sin\theta_{kd}\sin\theta_{km}] \end{array}$$

where the general and simplified result given in Ferreira et al. [19] as

(A50) $$\begin{array}{c} E\left[c_{i}^{2}s_{k}^{2}\right]=E\left[{\left(\sum_{l=1}^{N}\left|h_{il}^{LD}\right|\left|h_{l}^{SL}\right|\cos\theta_{il}\right)}^{2}{\left(\sum_{m=1}^{N}\left|h_{km}^{LD}\right|\left|h_{m}^{SL}\right|\sin\theta_{km}\right)}^{2}\right]\ldots \\ +E\left[{\left(\sum_{l=1}^{N}\left|h_{il}^{LD}\right|\left|h_{l}^{SL}\right|\cos\theta_{il}\right)}^{2}\right]\frac{\sigma_{SD}^{2}}{2}+E\left[{\left(\sum_{m=1}^{N}\left|h_{km}^{LD}\right|\left|h_{m}^{SL}\right|\sin\theta_{km}\right)}^{2}\right]\frac{\sigma_{SD}^{2}}{2}+\frac{\sigma_{SD}^{4}}{4} \end{array}$$

The in-phase term is

(A51) $$E\left[{\left(\sum_{l=1}^{N}\left|h_{il}^{LD}\right|\left|h_{l}^{SL}\right|\cos\theta_{il}\right)}^{2}\right]=E\left[\sum_{l=1}^{N}\sum_{t=1}^{N}\left|h_{il}^{LD}\right|\left|h_{it}^{LD}\right|\left|h_{l}^{SL}\right|\left|h_{m}^{SL}\right|\cos\theta_{il}\cos\theta_{it}\right]=\frac{N}{2}\chi_{LD}\chi_{SL}\left(1+\alpha_{2}\right)+N\left(N-1\right)\mu_{LD}^{2}\mu_{SL}^{2}\alpha_{1}^{2}$$

and the quadrature term is

(A52) $$E\left[{\left(\sum_{m=1}^{N}\left|h_{km}^{LD}\right|\left|h_{m}^{SL}\right|\sin\theta_{km}\right)}^{2}\right]=E\left[\sum_{d=1}^{N}\sum_{m=1}^{N}\left|h_{kd}^{LD}\right|\left|h_{km}^{LD}\right|\left|h_{d}^{SL}\right|\left|h_{m}^{SL}\right|\sin\theta_{kd}\sin\theta_{km}\right]=N\left(\chi_{LD}\chi_{SL}\frac{1}{2}\left(1-\alpha_{2}\right)\right)$$

where

(A53) $$\chi_{LD}=E\left[{\left|h_{il}^{LD}\right|}^{2}\right]=\Omega_{LD}$$

(A54) $$\chi_{SL}=E\left[{\left|h_{d}^{SL}\right|}^{2}\right]=\Omega_{SL}$$

(A55) $$\xi_{LD}=E\left[{\left|h_{il}^{LD}\right|}^{3}\right]=\frac{\Gamma (1.5+m_{LD})}{\left(\frac{m_{LD}}{\Omega_{LD}}\right)\Gamma \left(m_{LD}\right)}$$

(A56) $$\xi_{SL}=E\left[{\left|h_{d}^{SL}\right|}^{3}\right]=\frac{\Gamma (1.5+m_{SL})}{\left(\frac{m_{SL}}{\Omega_{SL}}\right)\Gamma \left(m_{SL}\right)}$$

(A57) $$\tau_{LD}=E\left[{\left|h_{il}^{LD}\right|}^{4}\right]=\frac{\left(1+m_{LD}\right)\Omega_{LD}^{2}}{m_{LD}}$$

(A58) $$\tau_{SL}=E\left[{\left|h_{d}^{SL}\right|}^{4}\right]=\frac{\left(1+m_{SL}\right)\Omega_{SL}^{2}}{m_{SL}}$$

The term $E[s_{i}^{2}s_{k}^{2}]$ can be easily obtained because $s_{i}$ and $s_{k}$ are uncorrelated and zero mean, then

(A59) $$E\left[s_{i}^{2}s_{k}^{2}\right]=\left\{\begin{array}{ll} {\left(E\left[s_{i}^{2}\right]\right)}^{2} & i\neq k\\ E\left[s_{k}^{4}\right]=3{\left(\mathrm{var}\left(s_{k}\right)\right)}^{2} & i=k \end{array}\right.$$

and, by substituting the variances; consequently, (A60).

(A60) $$E\left[s_{i}^{2}s_{k}^{2}\right]=\left\{\begin{array}{cc} \frac{1}{4}{\left(\sigma_{SD}^{2}+N\left(1-\alpha_{2}\right)\left(\sigma_{LD}^{2}\sigma_{SL}^{2}+\sigma_{LD}^{2}\mu_{SL}^{2}+\sigma_{SL}^{2}\mu_{LD}^{2}+\mu_{SL}^{2}\mu_{LD}^{2}\right)\right)}^{2} & i\neq k\\ \frac{3}{4}{\left(\sigma_{SD}^{2}+N\left(1-\alpha_{2}\right)\left(\sigma_{LD}^{2}\sigma_{SL}^{2}+\sigma_{LD}^{2}\mu_{SL}^{2}+\sigma_{SL}^{2}\mu_{LD}^{2}+\mu_{SL}^{2}\mu_{LD}^{2}\right)\right)}^{2} & i=k \end{array}\right.$$

Finally, the term $E[Z_{i}Z_{k}]$ can be calculated by using the formulas of $E[c_{i}^{2}c_{k}^{2}]$, $E[c_{i}^{2}s_{k}^{2}]$ and $E[s_{i}^{2}s_{k}^{2}]$, resulting in (A61).

(A61) $$\begin{array}{c} E\left[Z_{i}Z_{k}\right]=\left\{\begin{array}{ll} a_{1}^{4}\left(1-\frac{4}{N}\right)+a_{1}^{2}\left(\sigma_{SD}^{2}+a_{4}+Na_{2}a_{3}-\frac{2}{N}+4a_{5}\right)2a_{5}+\frac{1}{4}{\left(\sigma_{SD}^{2}+a_{4}+Na_{2}a_{3}\right)}^{2}+\\ \frac{1}{2}\left(N^{2}-N\right)\left(\chi_{LD}^{2}\chi_{SL}^{2}\left(1-\alpha_{2}^{2}\right)+4\mu_{LD}^{2}\chi_{LD}\mu_{SL}\xi_{SL}\alpha_{1}^{2}\left(1-\alpha_{2}\right)\right)+\frac{N}{2}\chi_{LD}^{2}\tau_{SL}\left(1-\alpha_{2}^{2}\right)\\ +\frac{1}{2}\sigma_{SD}^{2}\left(N\chi_{LD}\chi_{SL}\left(1+\alpha_{2}\right)+2\left(N-1\right)\mu_{LD}^{2}\mu_{SL}^{2}\alpha_{1}^{2}+\chi_{LD}\chi_{SL}\left(1-\alpha_{2}\right)\right)+\\ \left(N^{3}-3N^{2}+2N\right)\mu_{LD}^{2}\chi_{LD}\mu_{SL}^{2}\chi_{SL}\alpha_{1}^{2}\left(1-\alpha_{2}\right)+\frac{1}{2}\sigma_{SD}^{4}+\\ \frac{1}{4}{\left(\sigma_{SD}^{2}+N\left(1-\alpha_{2}\right)\left(\sigma_{LD}^{2}\sigma_{SL}^{2}+\sigma_{LD}^{2}\mu_{SL}^{2}+\sigma_{SL}^{2}\mu_{LD}^{2}+\mu_{SL}^{2}\mu_{LD}^{2}\right)\right)}^{2} & i\neq k\\ a_{1}^{4}+3a_{1}^{2}\left(\sigma_{SD}^{2}+a_{4}+Na_{2}a_{3}\right)+\frac{3}{4}\left(\sigma_{SD}^{2}+a_{4}+Na_{2}a_{3}\right)+\\ \frac{1}{4}\left(N^{2}-N\right)\left(\chi_{LD}^{2}\chi_{SL}^{2}\left(1-\alpha_{4}\right)+4\mu_{LD}\xi_{LD}\mu_{SL}\xi_{SL}\left(\alpha_{1}-\alpha_{3}\right)\alpha_{1}\right)+\frac{N}{2}\tau_{LD}\tau_{SL}\left(1-\alpha_{2}^{2}\right)+\\ \left(N^{3}-3N^{2}+2N\right)\mu_{LD}^{2}\chi_{LD}\mu_{SL}^{2}\chi_{SL}\alpha_{1}^{2}\left(1-\alpha_{2}\right)+\frac{N}{2}\sigma_{SD}^{2}[\chi_{LD}\chi_{SL}\left(1+\alpha_{2}\right)+\\ 2\left(N-1\right)\mu_{LD}^{2}\mu_{SL}^{2}\alpha_{1}^{2}+\chi_{LD}\chi_{SL}\left(1-\alpha_{2}\right)]+\\ \frac{1}{2}\sigma_{SD}^{4}+\frac{3}{4}{\left(\sigma_{SD}^{2}+N\left(1-\alpha_{2}\right)\left(\sigma_{LD}^{2}\sigma_{SL}^{2}+\sigma_{LD}^{2}\mu_{SL}^{2}+\sigma_{SL}^{2}\mu_{LD}^{2}+\mu_{SL}^{2}\mu_{LD}^{2}\right)\right)}^{2} & i=k \end{array}\right. \end{array}$$

The covariance $\mathrm{cov}(Z_{i}Z_{k})$ can be calculated by (A62) using the result of (A61). Since $\mathrm{var}\left(Z_{i}\right)=\mathrm{cov}\left(Z_{i},Z_{i}\right)$, the variance of the overall fading coefficient can be easily obtained by (A32) as shown in (A63):

(A62) $$\begin{array}{c} \mathrm{cov}\left(Z_{i}Z_{k}\right)=\left\{\begin{array}{cc} a_{1}^{4}\left(1-\frac{4}{N}\right)+a_{1}^{2}\left(\sigma_{SD}^{2}+a_{4}+Na_{2}a_{3}-\frac{2}{N}+4a_{5}\right)2a_{5}+\frac{1}{4}{\left(\sigma_{SD}^{2}+a_{4}+Na_{2}a_{3}\right)}^{2}+\\ \frac{1}{2}\left(N^{2}-N\right)\left(\chi_{LD}^{2}\chi_{SL}^{2}\left(1-\alpha_{2}^{2}\right)+4\mu_{LD}^{2}\chi_{LD}\mu_{SL}\xi_{SL}\alpha_{1}^{2}\left(1-\alpha_{2}\right)\right)+\frac{N}{2}\chi_{LD}^{2}\tau_{SL}\left(1-\alpha_{2}^{2}\right)\\ +\frac{1}{2}\sigma_{SD}^{2}\left(N\chi_{LD}\chi_{SL}\left(1+\alpha_{2}\right)+2\left(N-1\right)\mu_{LD}^{2}\mu_{SL}^{2}\alpha_{1}^{2}+\chi_{LD}\chi_{SL}\left(1-\alpha_{2}\right)\right)+\\ \left(N^{3}-3N^{2}+2N\right)\mu_{LD}^{2}\chi_{LD}\mu_{SL}^{2}\chi_{SL}\alpha_{1}^{2}\left(1-\alpha_{2}\right)+\frac{1}{2}\sigma_{SD}^{4}+\\ \frac{1}{4}{\left(\sigma_{SD}^{2}+N\left(1-\alpha_{2}\right)\left(\sigma_{LD}^{2}\sigma_{SL}^{2}+\sigma_{LD}^{2}\mu_{SL}^{2}+\sigma_{SL}^{2}\mu_{LD}^{2}+\mu_{SL}^{2}\mu_{LD}^{2}\right)\right)}^{2}\\ -\frac{1}{4}\times (2\sigma_{SD}^{2}+2N^{2}\times \mu_{LD}^{2}\;\mu_{SL}^{2}\;\alpha_{1}^{2}+\\ N[\left(1+\alpha_{2}\right)\times \mu_{SL}^{2}\mu_{LD}^{2}+\left(\sigma_{LD}^{2}\sigma_{SL}^{2}+\sigma_{LD}^{2}\mu_{SL}^{2}+\sigma_{SL}^{2}\mu_{LD}^{2}\right)\left(1+\alpha_{2}+2\alpha_{1}^{2}\right)]+\\ N\left(1-\alpha_{2}\right)\left(\sigma_{LD}^{2}\sigma_{SL}^{2}+\sigma_{LD}^{2}\mu_{SL}^{2}+\sigma_{SL}^{2}\mu_{LD}^{2}+\mu_{SL}^{2}\mu_{LD}^{2}\right){)}^{2} & i\neq k\\ a_{1}^{4}+3a_{1}^{2}\left(\sigma_{SD}^{2}+a_{4}+Na_{2}a_{3}\right)+\frac{3}{4}\left(\sigma_{SD}^{2}+a_{4}+Na_{2}a_{3}\right)+\\ \frac{1}{4}\left(N^{2}-N\right)\left(\chi_{LD}^{2}\chi_{SL}^{2}\left(1-\alpha_{4}\right)+4\mu_{LD}\xi_{LD}\mu_{SL}\xi_{SL}\left(\alpha_{1}-\alpha_{3}\right)\alpha_{1}\right)+\frac{N}{2}\tau_{LD}\tau_{SL}\left(1-\alpha_{2}^{2}\right)+\\ \left(N^{3}-3N^{2}+2N\right)\mu_{LD}^{2}\chi_{LD}\mu_{SL}^{2}\chi_{SL}\alpha_{1}^{2}\left(1-\alpha_{2}\right)+\frac{N}{2}\sigma_{SD}^{2}[\chi_{LD}\chi_{SL}\left(1+\alpha_{2}\right)+\\ 2\left(N-1\right)\mu_{LD}^{2}\mu_{SL}^{2}\alpha_{1}^{2}+\chi_{LD}\chi_{SL}\left(1-\alpha_{2}\right)]+\frac{1}{2}\sigma_{SD}^{4}+\\ \frac{3}{4}{\left(\sigma_{SD}^{2}+N\left(1-\alpha_{2}\right)\left(\sigma_{LD}^{2}\sigma_{SL}^{2}+\sigma_{LD}^{2}\mu_{SL}^{2}+\sigma_{SL}^{2}\mu_{LD}^{2}+\mu_{SL}^{2}\mu_{LD}^{2}\right)\right)}^{2}\\ -\frac{1}{4}\times (2\sigma_{SD}^{2}+2N^{2}\times \mu_{LD}^{2}\;\mu_{SL}^{2}\;\alpha_{1}^{2}+\ldots \\ N[\left(1+\alpha_{2}\right)\times \mu_{SL}^{2}\mu_{LD}^{2}+\ldots \left(\sigma_{LD}^{2}\sigma_{SL}^{2}+\sigma_{LD}^{2}\mu_{SL}^{2}+\sigma_{SL}^{2}\mu_{LD}^{2}\right)\left(1+\alpha_{2}+2\alpha_{1}^{2}\right)]+\\ N\left(1-\alpha_{2}\right)\left(\sigma_{LD}^{2}\sigma_{SL}^{2}+\sigma_{LD}^{2}\mu_{SL}^{2}+\sigma_{SL}^{2}\mu_{LD}^{2}+\mu_{SL}^{2}\mu_{LD}^{2}\right){)}^{2} & i=k \end{array}\right. \end{array}$$

(A63) $$\mathrm{var}\left(\gamma_{D}\right)=M\;\mathrm{var}\left(Z_{i}\right)+M\left(M-1\right)\;\;\mathrm{cov}\left(Z_{i},Z_{k}\right)$$

## References

[1] Boulogeorgos A.A.A., Alexiou A. (2020). Performance Analysis of Reconfigurable Intelligent Surface-Assisted Wireless Systems and Comparison with Relaying. IEEE Access.

[2] Wymeersch H., He J., Denis B., Clemente A., Juntti M. (2020). Radio Localization and Mapping with Reconfigurable Intelligent Surfaces: Challenges, Opportunities, and Research Directions. IEEE Veh. Technol. Mag..

[3] Yang Y., Zheng B., Zhang S., Zhang R. (2020). Intelligent Reflecting Surface Meets OFDM: Protocol Design and Rate Maximization. IEEE Trans. Commun..

[4] Basar E. Transmission through large intelligent surfaces: A new frontier in wireless communications. Proceedings of the 2019 European Conference on Networks and Communications (EuCNC).

[5] Perović N.S., Renzo M.D., Flanagan M.F. Channel Capacity Optimization Using Reconfigurable Intelligent Surfaces in Indoor mmWave Environments. Proceedings of the ICC 2020—2020 IEEE International Conference on Communications (ICC).

[6] Elbir A.M., Papazafeiropoulos A., Kourtessis P., Chatzinotas S. (2020). Deep Channel Learning for Large Intelligent Surfaces Aided mm-Wave Massive MIMO Systems. IEEE Wirel. Commun. Lett..

[7] Lin J., Wang G., Fan R., Tsiftsis T.A., Tellambura C. (2019). Channel Estimation for Wireless Communication Systems Assisted by Large Intelligent Surfaces. arXiv.

[8] Taha A., Alrabeiah M., Alkhateeb A. Deep learning for large intelligent surfaces in millimeter wave and massive MIMO systems. Proceedings of the 2019 IEEE Global Communications Conference (GLOBECOM).

[9] Hu S., Rusek F. Spherical Large Intelligent Surfaces. Proceedings of the ICASSP 2020—2020 IEEE International Conference on Acoustics, Speech and Signal Processing (ICASSP).

[10] Najafi M., Jamali V., Schober R., Poor V.H. (2020). Physics-based Modeling and Scalable Optimization of Large Intelligent Reflecting Surfaces. arXiv.

[11] Garcia J.C.B., Sibille A., Kamoun M. (2020). Reconfigurable Intelligent Surfaces: Bridging the Gap Between Scattering and Reflection. IEEE J. Sel. Areas Commun..

[12] Kishk M.A., Alouini M.S. (2020). Exploiting Randomly-located Blockages for Large-Scale Deployment of Intelligent Surfaces. arXiv.

[13] Mukherjee M., Kumar V., Guo M., da Costa D.B., Basar E., Ding Z. (2021). The Interplay of Reconfigurable Intelligent Surfaces and Mobile Edge Computing in Future Wireless Networks: A Win-Win Strategy to 6G. arXiv.

[14] Malandrino F., Nordio A., Chiasserini C.F. (2021). Eavesdropping with Intelligent Reflective Surfaces: Threats and Defense Strategies. arXiv.

[15] Yang L., Yang J., Xie W., Hasna M.O., Tsiftsis T., Renzo M.D. (2020). Secrecy Performance Analysis of RIS-Aided Wireless Communication Systems. IEEE Trans. Veh. Technol..

[16] Trigui I., Agbogla E.K., Benjillali M., Ajib W., Zhu W.P. (2021). Bit Error Rate Analysis for Reconfigurable Intelligent Surfaces with Phase Errors. arXiv.

[17] Ai Y., de Figueiredo F.A.P., Kong L., Cheffena M., Chatzinotas S., Ottersten B. (2021). Secure Vehicular Communications Through Reconfigurable Intelligent Surfaces. IEEE Trans. Veh. Technol..

[18] Makarfi A.U., Rabie K.M., Kaiwartya O., Adhikari K., Li X., Quiroz-Castellanos M., Kharel R. (2020). Reconfigurable Intelligent Surfaces-Enabled Vehicular Networks: A Physical Layer Security Perspective. arXiv.

[19] Coelho Ferreira R., Facina M.S., de Figueiredo F.A., Fraidenraich G., de Lima E.R. (2020). Large Intelligent Surfaces Communicating through Massive MIMO Rayleigh Fading Channels. Sensors.

[20] Best D., Fisher N.I. (1979). Efficient simulation of the von Mises distribution. J. R. Stat. Soc. Ser. (Appl. Stat.).

[21] Beran R. (1977). Minimum Hellinger distance estimates for parametric models. Ann. Stat..

[22] Brychkov Y.A., Marichev O., Prudnikov A. (1988). Integrals and Series: Special Functions.

[23] Proakis J.G. (2007). Digital Communications.

[24] Lin S.H., Lu R.R., Fu X.T., Tong A.L., Wang J.Y. (2019). Physical-Layer Security Analysis over M-Distributed Fading Channels. Entropy.

